# CD44 Receptor-Mediated Ferroptosis Induction by Hyaluronic Acid Carbon Quantum Dots in Triple-Negative Breast Cancer Cells Through Downregulation of SLC7A11 Pathway

**DOI:** 10.3390/ma18092139

**Published:** 2025-05-06

**Authors:** Karthikeyan Chandrasekaran, Chae Eun Lee, Seojeong Yun, Ashok Kumar Jangid, Sungjun Kim, Kyobum Kim

**Affiliations:** Department of Chemical & Biochemical Engineering, Dongguk University, Seoul 04620, Republic of Korea; karthiisro@gmail.com (K.C.); jum0051@dgu.ac.kr (C.E.L.); yunsj9682@gmail.com (S.Y.); ashok4483@gmail.com (A.K.J.); sungjun.kim@dgu.ac.kr (S.K.)

**Keywords:** hyaluronic acid (HA), carbon quantum dots (CQDs), reactive oxygen species (ROS), glutathione depletion, lipid peroxidation, ferroptosis

## Abstract

The field of cancer therapy is actively pursuing highly effective self-targeted drug delivery materials endowed with exceptional properties. Recently, hyaluronic acid (HA), a naturally occurring polysaccharide, has been recognized as a potential target ligand for CD44 receptors, which are frequently expressed on various solid tumor cells targeted in cancer therapy. HA carbon quantum dots (CQDs) exhibit several advantageous properties, including a high surface area-to-volume ratio, small particle size, biocompatibility, and low cytotoxicity, making them ideal for biomedical applications, such as CD44-targeted drug delivery in ferroptosis-based cancer therapy. In this study, we synthesized HA-CQDs to enhance CD44-mediated ligand–receptor interactions targeting triple-negative breast cancer (TNBC). CQDs facilitate the intracellular generation of reactive oxygen species (ROS), leading to glutathione depletion. These processes result in crucial actions such as the downregulation of glutathione peroxidase 4, downregulation of solute carrier family 7 member 11, and inhibition of cystine intake. The subsequent intracellular ROS, originating from lipid peroxidation, induces ferroptosis. Our HA-CQDs engage CD44 receptors, selectively targeting TNBCs and enhancing cancer recognition. This interaction potentially enhances the nanoplatform-based CD44 targeted therapeutic effects in inducing ferroptosis.

## 1. Introduction

Ferroptosis represents a distinctive form of non-apoptotic programmed cell death, characterized by morphological, genetic, and biochemical distinctions from other regulated cell death modalities [1]. Biochemically, the depletion of intracellular glutathione (GSH) [2] leads to the suppression of glutathione peroxidase 4 (GPX4), resulting in an accumulation of reactive oxygen species (ROS) from lipid peroxides, which drive ferroptosis [3]. Genetic modifications, such as changes in iron homeostasis and lipid peroxidation metabolism, also contribute to this process [4]. Various nanomaterials, including carbon quantum dots, induce ferroptosis [5,6]. These materials trigger ferroptosis by reducing GSH levels, achieved through the direct inhibition of system Xc-, an amino acid antiporter prevalent in the phospholipid bilayer. System Xc- subunits, such as solute carrier family 3 member 2 and solute carrier family 7 member 11 (SLC7A11), form heterodimers crucial for cellular antioxidant defense [7]. System Xc- facilitates the exchange of glutamate for cystine at a 1:1 ratio. Cystine is then utilized to synthesize GSH, while GPX4 controls ROS levels [8]. Inhibiting system Xc- activity impairs GSH biosynthesis by blocking cystine uptake, subsequently reducing GPX4 activity, compromising cellular antioxidant capacity, and promoting lipid ROS accumulation, ultimately leading to oxidative damage in cytoplasmic membranes. Additionally, the downregulation of SLC7A11 expression and inactivation of GPX4 function further inhibits cellular antioxidant capability and enhances lipid ROS buildup, ultimately driving ferroptosis [9].

In recent decades, carbon quantum dots (CQDs) have garnered significant attention in the biomedical industry for their advantages, such as water dispersibility, luminescence, biocompatibility, and cost-effective synthesis [10,11]. The small size of CQDs, less than 10 nm, challenges their accumulation at tumor sites due to enhanced permeability and retention effects and limitations imposed by nanoparticle size (20–200 nm) [12]. Imaging and cancer treatments impact the decreased concentration of CQDs at tumor sites [13]. CQDs exhibit nonspecific biodistribution with normal tissues, leading to toxicity in healthy cells [11]. CQD-based diagnostic and therapeutic applications aim to deliver CQDs primarily to tumor sites to mitigate this issue [13]. CQD-synthesized precursors are utilized as self-targeted strategy materials, enhancing the delivery of CQD-based nanoplatforms into tumor sites and facilitating the identification of tumor-targeted specificity [14]. In this study, hyaluronic acid (HA) was selected for CQD preparation due to its properties as a water-soluble polysaccharide and its high affinity for binding the surface marker cluster for cancer cells, such as the CD44 receptor [15,16]. HA is a naturally occurring polysaccharide, a glycosaminoglycan with an exceptionally high molecular weight. It consists of regularly repeating units of N-acetyl-D-glucosamine and D-glucuronic acid [17,18]. These units polymerize into large macromolecules comprising over 60,000 repeating units. HA molecules are abundantly present in the extracellular matrix of articular cartilage and play a crucial role in various cellular processes [15].

**Figure 1:**
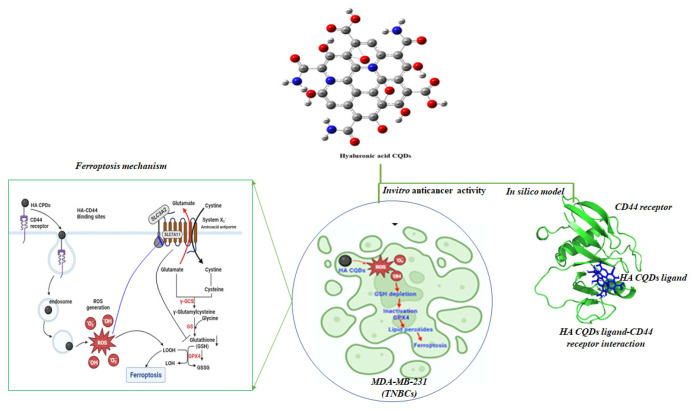
Schematic diagram illustrating HA-CQD-mediated ferroptosis-based CD44-targeted anticancer activity in triple-negative breast cancer cells.

We synthesized HA-CQDs using a one-step hydrothermal synthesis method. The prepared HA-CQD surface actively targets CD44-positive cancer cells. To investigate the specific binding affinity of CD44-mediated endocytosis, three different cell types were selected for cytotoxicity analysis: one normal fibroblast (low expression) and two different cancer cells, including triple-negative breast cancer (MDA-MB-231, CD44 positive) and liver cancer cells (HepG2, CD44 negative). We demonstrated cancer cell death based on the following: (1) cell death triggered by HA-CQDs through ferroptosis-based CD44-mediated endocytosis, (2) ferroptosis induction by HA-CQDs, marked by the assessment of ROS, depletion of GSH, downregulation of SLC7A11, downregulation of GPX4, and upregulation of lipid peroxidation. We hypothesized that modifying the HA-CQDs would enhance their natural binding affinity to the CD44 receptor. The interactions between the HA-CQD ligand and the CD44 receptor were assessed using in silico approaches, specifically molecular docking analysis. The schematic diagram (Figure 1) illustrates HA-CQDs facilitating ferroptosis-based CD44 targeted anticancer activity in triple-negative breast cancer cells.

## 2. Materials and Methods

### 2.1. Materials

HA (MW 60 kDa) was obtained from Life-core Biomedical (Chaska, MN, USA), and deuterated D2O NMR solvent was procured from Sigma-Aldrich (St. Louis, MO, USA).

### 2.2. HA-CQDs Synthesis

HA-CQDs were synthesized via a one-pot hydrothermal method. Briefly, 100 mg of HA was dissolved in 60 mL of distilled water (with a resistivity of 9.21 mΩ-cm), transferred to a 100 mL autoclave (Teflon vessel covered with Autoclave Reactor w/PTFE Lined 250 mL stainless steel), and carbonized at 180 °C for 12 h in a hot air oven. The resulting dark brown solution was then filtered through a 0.25 μm membrane micro-filter. The filtered HA-CQD solution underwent dialysis in distilled water and was further purified using a 1 KDa cellulose dialysis membrane for 48 h. This purification removed impurities and redundant precursors, enhancing the stability of the HA-CQD solutions and allowing for the isolation of HA-CQDs with the desired size. The HACQD solutions were freeze-dried for 48 h (Sunil Trading, Seoul, Republic of Korea), yielding highly pure HA-CQDs, which were stored in a refrigerator (4 °C) until further use. Figure 1 illustrates the synthesis process of HA-CQDs.

### 2.3. Characterization Analysis

TEM micrographs of HA CQDs were captured using a Tecnai G2 spirit twin microscope (FEI, Lausanne, Switzerland) with an accelerating voltage of 120 kV. The surface area of the nanosized material was calculated through Brunauer–Emmett–Teller (BET) analysis. N2 adsorption–desorption experiments were conducted at 77.350 K using Quantachrome Instruments v11.05 (Quantachrome, NovaWin, Boynton Beach, FL, USA). Raman spectra of the films were recorded using a micro-Raman spectrometer (Acton SpectraPro 2500i, Princeton Instruments, Acton Optics & Coatings, Acton, MA, USA) with an Ar laser source emitting at a wavelength of 514.5 nm. The structure of HA CQDs was confirmed through 1H-NMR analysis (500 MHz FT-NMR spectrometer, Bruker, Bremen, Germany) and FTIR analysis (PerkinElmer FTIR Spectrum Two, PerkinElmer, Springfield, IL, USA). Fourier transform infrared spectroscopy (FTIR) spectra were acquired using a PerkinElmer FTIR Spectrum Two spectrometer, covering the range of 4000–600 cm^−1^. XPS measurements were conducted using an XPS instrument from Carl Zeiss, Oberkochen, Germany. Room temperature UV/vis/NIR absorption spectra were recorded using a V-770 spectrophotometer (JASCO, Tokyo, Japan). The NIR-II fluorescence spectra of HA CQD were collected at room temperature using a FluoroMax Plus spectrofluorometer (Horiba, Tokyo, Japan) with excitation laser sources emitting at 450 nm (PL) and 808 nm (PLQY).

### 2.4. Molecular Docking Study

Ligand preparation: The ligand molecules were initially sketched and transformed from 2D to 3D using Marvin Sketch. Subsequently, these molecules were processed and converted into the PDBQT file format using AutoDock Tools (ADT).

Protein preparation: The receptor structure of the murine CD44 hyaluronan binding domain (PDB ID:4MRD, Resolution: 2.55 Å) was retrieved from the protein data bank. Protein molecules were prepared by removing water molecules and cofactors and adding polar H bonds and charges using ADT.

Molecular docking: Molecular docking of hyaluronic acid and HA CQD ligand molecules with the murine CD44 hyaluronan binding domain was conducted using AutoDock Vina. The protein’s active site was identified through the Computed Atlas of Surface Topography of Proteins (CASTp) server. CASTp server predicted the best binding pockets with a high surface area (164.306 Å^2^) and volume (133.109 Å^3^) as the active sites for the protein. The amino acids in the active site of the protein were identified as ASN29, VAL30, THR31, CYS32, TYR34, HIS39, GLU41, GLY77, PHE78, GLY79, THR80, CYS81, ARG82, VAL153, ASN154, and ARG155 (Table S1). A grid box was generated using ADT with dimensions relative to the ligands (20 Å × 20 Å × 20 Å) with a resolution of 1 Å. Gasteiger charges and polar hydrogens were added to CD44, and hyaluronic acid and HA-CQDs were modified files submitted to AutoDock Vina V1.1.2. Each docking calculation was repeated three times using different seeds and retaining the remaining values as default. Final protein–ligand interactive models were chosen based on the binding affinity and the molecular contacts. Protein–ligand interaction profiler (PLIP) was used to determine H-bonds and non-bonded interactions. Three-dimensional stereo figures of protein–ligand interactions were computed using PyMOL Version 3.1. Table S1 showed CD44 (4MRD) protein amino acid position parameters.

### 2.5. Anticancer Effect of HA CQDs

The cell viability was determined by WST-1 assay (DoGenBio, Seoul, Republic of Korea) and live and dead (L/D) staining. MDA-MB-231, HepG2, and Fibroblast cells were seeded to 96 well plates at a density of 10,000 cells/well and incubated overnight. And 100 uL of HA CQDs with different concentrations of HA-CQDs (25, 50, 75, 100, 125, and 150 μg·mL^−1^) were treated to cells for 24 h at 37 °C. For L/D staining, 100 μL of L&D solution containing 2 μM of Calcein-AM and 4 μM of ethidium homodimer was added to the wells, and the cells were stained for 30 min at 37 °C (in the dark). After washing, images were observed using fluorescence microscopy (Nikon TI−E, Tokyo, Japan). For WST-1, an EZ-Cytox assay kit (DoGenBio, Seoul, Republic of Korea) was used according to the manufacturer’s protocol. WST-1 reagent (EZ-cytox:cell culture media = 1:10) was added to the wells and incubated for 3 h in the dark. The absorbance was measured using a microplate reader at a wavelength of 450 nm (Spectra Max ID3, Molecular Devices, San Jose, CA, USA). Cell viability was calculated with the following formula:

Cell viability (%) = (Experiment group-Blank)/(Control group-Blank) × 100

### 2.6. Intracellular ROS Generation

The MDA-MB-231 and HepG2 cells were cultured in Dulbecco’s Modified Eagle’s Medium (DMEM, Corning, Somerville, MA, USA) containing 10% Fetal Bovine Serum (FBS, Corning, Somerville, MA, USA) and 1% Penicillin Streptomycin (P/S, Corning, Somerville, MA, USA). MDA-MB-231 and HepG2 cells were seeded to 96 well plates at a density of 10,000 cells/well and incubated overnight. MDA-MB-231 cells were incubated with different concentrations of HA CQDs (50 and 100 μg·mL^−1^) in DMEM for 6 h. After that, a DCFH-DA solution (25 μM) was added to cells for 30 min at 37 °C in the dark. 2’,7’-dichlorodihydrofluorescein diacetate (DCFH-DA, Sigma-Aldrich, St. Louis, MO, USA) was used to detect intracellular ROS. Then, H_2_O_2_ (50 μM) was treated for 1 h as a positive control. After washing with 1× Dulbecco’s phosphate-buffered saline (DPBS, Corning, Somerville, MA, USA), the fluorescence intensity (F.I.) of DCFH was detected using a microplate reader (iD3, SpectraMax, San Jose, CA, USA). The fluorescence was obtained at 485 nm (excitation) and 535 nm (emission). Images were captured using fluorescence microscopy (Nikon TI−E, Tokyo, Japan).

### 2.7. Ellman’s Analysis for Intracellular GSH Content

The glutathione/glutathione disulfide (GSH/GSSG) system is crucial for cellular redox balance. Glutathione (GSH), a tripeptide (γ-glutamyl-cysteinyl-glycine), is the most abundant intracellular free thiol in eukaryotic cells. It plays a significant role in maintaining intracellular redox status and defending against oxidative stress. During oxidative stress, reduced GSH is oxidized to form glutathione disulfide (GSSG). Typically, cells maintain a high ratio of GSH to GSSG. Over 90% of total GSH is kept in its reduced form through de novo GSH synthesis, enzymatic reduction of GSSG, or uptake of exogenous GSH. A shift in the cellular GSH/GSSG ratio serves as a vital signal that can determine the fate of a cell. MDA-MB-231 and HepG2 cells were seeded to 6 well plates at a density of 300,000 cells/well and incubated overnight. MDA-MB-231 and HepG2 cells were incubated with different concentrations of HA-CQDs (50 and 100 μg·mL^−1^) in DMEM for 24 h at 37 °C. A total of 1 × 10^6^ cells/200 uL of cell suspension with 5% metaphosphoric acid solution was homogenized (20 s/rest 10 s) on ice. Cell lysate was centrifugated at 12,000 rpm for 10 min at 4 °C and supernatant was used. After that, GSH and GSSG samples were prepared and quantified using an EZ-glutathione assay kit (DoGen Bio) according to the manufacturer’s protocol. Then, the optical density (OD) was monitored at 412 nm by a microplate reader (iD3, Spectra Max). Ellman’s reagent (5,5’-dithiobis-2-nitrobenzoic acid, DTNB) (Sigma-Aldrich, St. Louis, MO, USA) was used to detect the GSH and GSSG. The GSH/GSSG ratio was calculated by the following formula:

GSH/GSSG ratio = (GSH_t_-2 GSSG)/GSSG

### 2.8. mRNA Expression of Ferroptosis Markers

Real-time-polymer chain reaction (RT-PCR) was conducted to confirm the ferroptosis-related genes such as glutathione peroxidase 4 (GPX4), solute carrier family 7 member 11 (SLC7A11), and long-chain-fatty-acid-CoA ligase 4 (ACSL4). Glyceraldehyde 3-phosphate dehydrogenase (GAPDH) was used as a housekeeping gene. Primer sequences for RT-PCR are listed in Table S2. After treating HA-CQDs of 0, 50, 100, and 125 ug/mL to cells, RNA of treated MDA-MB-231 and HepG2 cells were isolated using Trizol (Favorgen, Pingtung, Taiwan) and cDNAs were synthesized using ReverTra Ace qPCR RT Master Mix (Toyobo, Osaka, Japan). RT-PCR was then performed using a SYBR green master mix (Toyobo, Osaka, Japan) and a Step One Plus Real Time PCR System (Applied Biosystems, Foster City, CA, USA). Results were analyzed using the 2^−ΔΔCt^ method. Table S2 indicates the primer sequence for RT-PCR.

### 2.9. Lipid Peroxidation

In the initial stage, the HA-CQDs generate high levels of ROS and trigger the release of PUFAs. Lipid peroxidation of (polyunsaturated fatty acids) PUFAs produces a wide variety of oxidation products. Lipid hydroperoxides (L-OOHs) are the initial products of peroxidation. The process is to be terminated by producing secondary product electrophilic lipid aldehydes, such as 4-hydroxynonenal (4HNE), malondialdehyde (MDA), and acrolein (ACR). These aldehydes are with different proteins in the mitochondrial electron transport, leading to an electron flux, resulting in electrons reacting with O_2_ and more generation of ROS. MDA is a component that can decompose arachidonic acids (AAs) and larger PUFAs identified via enzymatic and nonenzymatic processes. MDA is the most mutagenic product of lipid peroxidation due to its ability to react with primary amines on proteins and DNA, forming crosslinked bonds. An excess amount of MDA generated within the cell can be associated with various disorders, including Alzheimer’s disease, cancer, heart disease, diabetes, and Parkinson’s disease. The decomposition of AAs and larger PUFA generates (4-hydroxynonenal) 4-HNE, which is a highly reactive product of lipid peroxidation and contains three functional groups: (1) an electrophilic C=C double bond is a Michael acceptor, forming covalent bonds with nucleophilic amino acids, (2) aldehydes can form Schiff base bonds with primary amines, and (3) hydroxyl groups can be oxidized to form electrophilic ketones. These aldehydes can be formed as a secondary product during lipid peroxidation. MDA has been widely used as a convenient biomarker for lipid peroxidation of omega-3 and omega-6 fatty acids because of their reaction with thio-barbituric acid (TBA). The TBA test is based on the reactivity of TBA to MDA and can indicate the degree of lipid peroxidation (Figure S1).

MDA-MB-231 and HepG2 cells were seeded to 6 well plates at 300,000 cells/well density and incubated overnight. Cells were incubated with different concentrations of HA-CQDs (0, 50, and 100 μg·mL^−1^) in DMEM for 24 h at 37 °C. Cells were suspended in PBS with 1X BHT and then homogenized (20 s/rest 10 s) on ice. After that, the MDA sample was prepared and quantified using an EZ-Lipid peroxidation (TBARS) assay kit (DoGenBio) according to the manufacturer’s protocol. The indicator solution was added to the standard and samples and reacted for 45 min at 65 °C. Then, the optical density (OD) was monitored at 540 nm by a microplate reader (iD3, Spectra-Max).

### 2.10. Statistical Analysis

GraphPad Prism 7.0 (GraphPad Software Inc., La Jolla, CA, USA) was used to conduct statistical analysis. All quantitative data are expressed as the mean ± standard deviation. In the case of quantitative data, experiments were carried out in triplicate (*n* = 3) and assessed using one-way analysis of variance (ANOVA) and Tukey’s multiple comparison test. Differences were considered statistically significant at *p* values of <0.05.

## 3. Results and Discussion

### 3.1. Formation Mechanism of HA Carbon Quantum Dots

**Figure 2:**
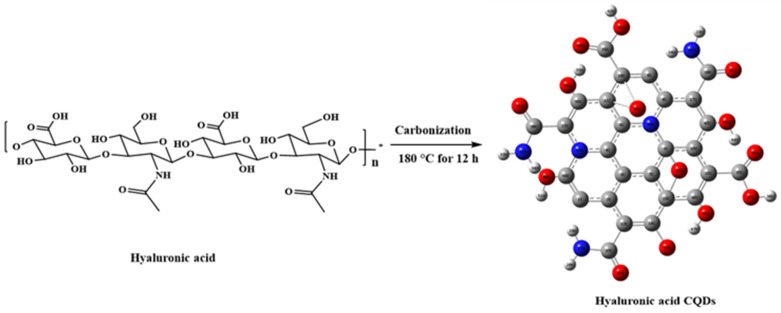
Synthesis procedure of hyaluronic acid carbon quantum dots.

CQDs, gaining increased attention in biomedical applications such as biosensing, bioimaging, and biotherapy [19], are often conjugated with active targeting agents to enhance specificity and delivery precision in CQD-based theragnostic platforms [20]. However, this approach involves multiple steps and may introduce complexity and potential interferences among components. To address these challenges, self-targeting molecules such as carbon precursors are utilized, endowing CQDs with inherent self-targeting capabilities [20]. One example is the biopolymer HA, which demonstrates a strong affinity for CD44 receptors on cell surfaces [21]. CD44 receptors are commonly overexpressed in various tumors, including breast, lung, and hepatocellular carcinomas. In this study, HA was used as a solute to synthesize CQDs. The synthesis pathway for HA-CQDs is illustrated in Figure 2. HA-CQDs were prepared via one-pot hydrothermal treatment at 180 °C for 12 h, using HA solely as the carbon source. During this process, the HA precursors underwent polymerization, dehydration, and carbonization, and ultimately formed CQDs. The resulting HA-CQDs displayed considerable stability in aqueous solutions and showed a graphitic carbon structure with some defects. Various functional groups found in the HA-CQDs, such as NHCO-, CO-, C-O-C, O-H, and COO-, reflect those in bulk HA.

### 3.2. TEM and BET Analysis

**Figure 3:**
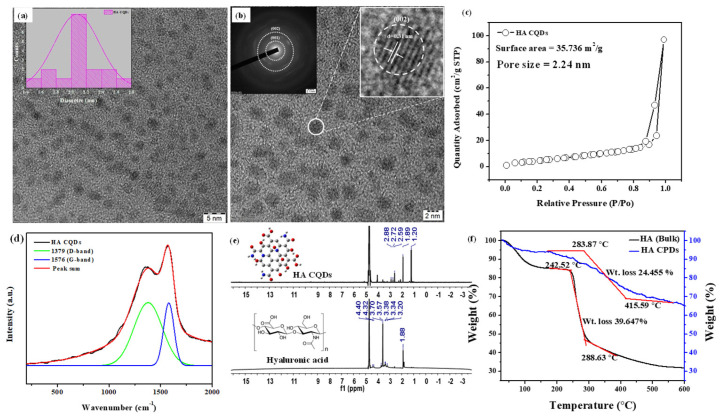
Characterization of HA-CQDs. (**a**,**b**) TEM and SEAD patterns of HA-CQDs, respectively; (**c**) BET analysis of HA-CQDs; (**d**) Micro Raman spectra of HA-CQDs; (**e**) H-1 NMR spectra; and (**f**) TGA analysis of HA and HA-CQDs. The red line indicates the thermal decomposition of the HA sample, which occurs in two distinct stages.

The TEM image of synthesized HA-CQDs is shown in (Figure 3a,b). The synthesized HA CQD exhibits a spherical structure with a diameter ranging from 1.4 to 2.8 nm. The average diameter of HA-CQDs is 2.1 nm, as determined by ImageJ software 1.53. These observations indicate that HA-CQDs form uniform nanodots with consistent size distribution. Figure 3b displays the SAED of the HA-CQD, with two dotted rings indicating particle formation at two bright spots, confirming their crystalline nature. Figure 3b also reveals that the HA-CQD has a crystal lattice distance of 0.31 nm, attributed to graphitic carbon (002) lattice spacing. The surface area and porosity of HA-CQDs were investigated by measuring nitrogen adsorption–desorption isotherms in Figure 3c at 77.3 K and characterized through BET analysis. According to the IUPAC classification [22], the observed loop can be attributed to type H3 hysteresis loops, indicating the presence of abundant pores. The average pore diameter measures approximately 2.24 nm. This smaller size enhances the BET surface area of the HA-CQDs, which is determined to be 35.736 m^2^/g. The increased surface area provides a large number of adsorption sites. Simultaneously, the increased number of pores and larger sample volume facilitate contact with target cells, enhancing their anticancer performance [23].

### 3.3. Micro Raman Analysis, NMR, and TGA Analysis

Micro Raman spectra analysis determined the structural hybridization (carbon) of the synthesized HA-CQDs, as illustrated in Figure 3d. The Raman spectrum revealed two peaks: a disordered (D) peak at 1379 cm^−1^ and a graphite (G) peak at 1576 cm^−1^. The D band originates from the vibrations of carbon atoms in disordered graphite while the G band corresponds to the E2g mode of structured graphite [24]. The analysis shows that the HA-CQDs comprise both sp3 hybrid orbitals (indicating carbon defects) and sp2 hybrid orbitals (indicative of a graphitic carbon structure), which together form the structure of the Carbon dots. The intensity ratio of ID/IG for the HA-CQDs is approximately 0.875, serving as an essential parameter for characterizing the CQDs’ structure and providing direct evidence of functionalization. Furthermore, the HA-CQDs possess various functional groups, including -OH, -COOH, -CO, and -NH2, contributing to their superior water disposability [25].

HA is a linear polysaccharide with the repeating disaccharide unit poly[(1→3)-β-d-GlcNAc-(1→4)-β-d-GlcA−] [26]. The 1H-NMR spectra of bulk HA (Figure 3e) displayed characteristic peaks for methyl protons of N-acetyl glucosamine (N-(CH3COCH3: δ 1.88 ppm), CH2 groups in HA units (δ 3.20, 3.34, 3.57, and 3.70 ppm) from sugar rings, the primary hydroxyl group (δ 4.36 ppm), and secondary hydroxyl groups connected to the ring (δ 4.45 ppm). Hyaluronic acid was carbonized at 180 °C, effectively converting it into HA CQD. The HA-CQDs showed peaks at (δ 1.2–1.88 ppm), (δ 2.59–2.88 ppm), and (δ 3.5 and 4.3 ppm), associated with the transition from sp3 to sp2hybridized carbon atoms and the presence of oxygen-containing groups (alcohols, carboxylic acids).

Thermal gravimetric analysis (TGA) was employed to assess the thermal stability of HA and HA-CQDs (Figure 3f) in a nitrogen atmosphere from 25 to 600 °C at a heating rate of 20 °C/min. The HA sample undergoes thermal decomposition in two stages: initially, the hydroxyl groups decompose, resulting in approximately 15% weight loss at 240 °C; subsequently, polysaccharide groups decompose, leading to a 39.647% weight loss between 240.52 °C and 288.63 °C. The total weight loss observed for HA is 68% at 600 °C.

The decomposition of HA-CQDs results in a weight loss of up to 35% within the 25–600 °C temperature range. In contrast, considering pure HA, the total weight loss amounts to 68% over the same temperature range. This considerable difference in weight loss highlights the superior thermal stability of HA-CQDs compared to their precursor, HA. The enhanced thermal stability of HA-CQDs is attributed to a transformation process involving the carbonization of HA at 180 °C for 12 h. During the phase transformation from bulk HA to HA-CQDs, the as-synthesized HA-CQDs retain the original monomer unit of HA, confirmed by FTIR analysis. However, HA-CQDs exhibit greater stability at high temperatures than HA because they adopt a graphite-like structure. This structural resemblance endows HA-CQDs with unique properties, such as targeting cancer cells via CD44 positive interaction—the HA-CD44 binding interaction downregulates cytoskeletal pathways involved in cancer cell death [27].

**Figure 4:**
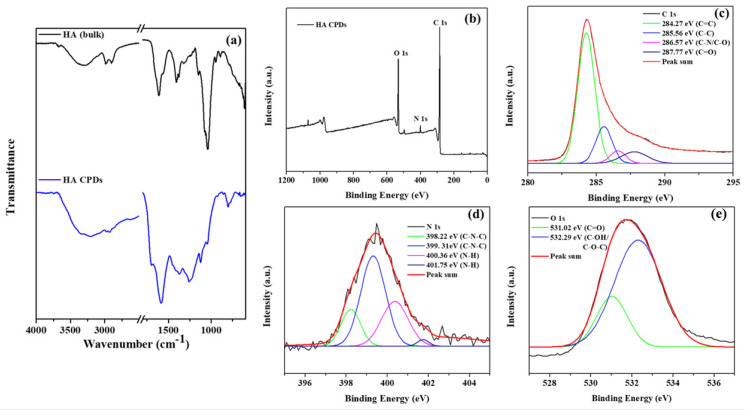
(**a**) FTIR spectra of HA and HA-CQDs; XPS spectra of HA-CQDs depict (**a**) wide scan, (**b**) C (1s) state, (**c**) C (1s) state, (**d**) N (1s) state, and (**e**) O (1s) state.

The FTIR spectra of HA and HA-CQDs are depicted in (Figure 4a), spanning a range from a minimal wavenumber of 600 cm^−1^ to a maximal wavelength of 4000 cm^−1^. A satisfactory fit using Gaussian convolution was achieved for both HA and HA-CQDs in the FTIR spectra of the samples (Figure S2(a1,a2)), along with the vibrational assignment of HA and HA-CQDs in Table S3. Solid lines in the figures signify the linear combination of various functional groups represented by Gaussian peaks. The HA spectrum reveals several prominent features, including broad peaks at 3670, 3545, 3279, and 3075 cm^−1^ corresponding to OH and -NH stretching vibrations [28]. Both asymmetric and symmetric stretching vibrations of sp3 hydrocarbon (C-H) groups are noted at 2981, 2900, and 2861 cm^−1^. Pronounced carboxylic (C=O) and amide (N-H) stretching are distinct at 1626 and 1566 cm^−1^, respectively. Stretching bands for N-acetyl (-COCH3) are observed at 1444 and 1406 cm^−1^ [29]. Additionally, C-H bending, and C-N stretching are clearly noted at 1374 and 1231 cm^−1^, respectively. The glycosidic ether (C-O-C) and alcoholic (C-OH) stretching features are detected at 1147 cm^−1^ and 1071/1031 cm^−1^, [28] respectively. The proteoglycan sugar ring (C-O) group is identified at 995, 950, and 929 cm^−1^. Additionally, C-H bending peaks occur at 868, 792, 742, 685, and 626 cm^−1^.

The FT-IR spectra of the HA-CQDs are depicted in (Figure S2(b1,b2)). The broad characteristic absorption peaks for N–H/O–H stretching vibrations are observed at 3950, 3441, 3194, and 3039 cm^−1^. Additional peaks appear at approximately 2968, 2925, and 2886 cm^−1^; 1587 cm^−1^; 1465 and 1427 cm^−1^; 1356 cm^−1^, 1249 cm^−1^; 1176 and 1116 cm^−1^; and 1091 and 1050 cm^−1^, along with 982 cm^−1^, corresponding to C-H stretching; N-H amide; N-acetyl (-COCH3) stretching; C-H bending; C-N stretching, glycosidic (ether) (C-O-C) group; alcoholic (C-OH) group; and the proteoglycan sugar ring (C-O) vibrations, respectively [25]. However, when HA bulk is converted into HA-CQDs, a significant shift in the carboxylic group occurs, moving from 1626 to 1701 cm^−1^. This transformation reflects the conversion of HA into HA carbon quantum dots. Furthermore, two new peaks emerge at 2754 and 2624 cm^−1^, attributed to the aldehyde (C-H) stretching [25]. C-H bending is also evident at 799 and 761 cm^−1^. HA-CQDs display functional groups similar to HA (–NHCO–, C=O, C–O–C, C-H, OH-, and COO- groups) [25] (Figure S1), retaining the original structure of HA. Optical studies such as the UV-Vis absorbance spectrum and the Photoluminescence spectrum of HA-CQDs are shown in Figure S3a–d.

### 3.4. X-Ray Photoelectron Spectroscopy (XPS) Studies

The elemental composition and oxidation states of HA-CQDs were determined using XPS, as illustrated in (Figure 4b–e). The elemental composition of HA-CQDs revealed 66.79% carbon, 17.71% oxygen, and 2.95% nitrogen. In Figure 4b, the XPS wide scan spectra of HA-CQDs exhibited three peaks at 285.08 eV, 532.22 eV, and 400.04 eV, respectively, associated with the oxidation states of C 1s, O 1s, and N 1s. The high-resolution spectrum of the C 1s oxidation state was resolved into four distinct binding energy signals at 284.27 eV (C=C), 285.56 eV (C-C), 286.57 eV (C-N/C-O), and 287.77 eV (C=O), indicative of sp2 and sp3 hybridization of carbon atoms, resembling a graphite structure [25]. The oxidation state of N 1s yielded four signals at 398.22 eV, 399.31 eV, 400.36 eV, and 401.75 eV, respectively; the signals at 398.22 eV and 399.31 eV corresponded to (C-N-C), while those at 400.36 eV and 401.75 eV corresponded to (N-H) groups. The oxidation state of O 1s generated two signals at 531.02 eV and 532.29 eV, assigned to the carboxyl group (C=O) and the carbonyl group (C-OH/C-O-C), respectively. These findings suggest the presence of aromatic structures, electron donors, and electron acceptor groups in the HA-CQDs [25]. Supported by the FTIR spectrum, these results demonstrate the formation of D-A (donor-acceptor) and D-Π-A (donor-pi-acceptor) structures, considerably enhancing the anticancer properties of HA-CQDs.

### 3.5. Molecular Docking of HA and HA-CQDs

**Figure 5:**
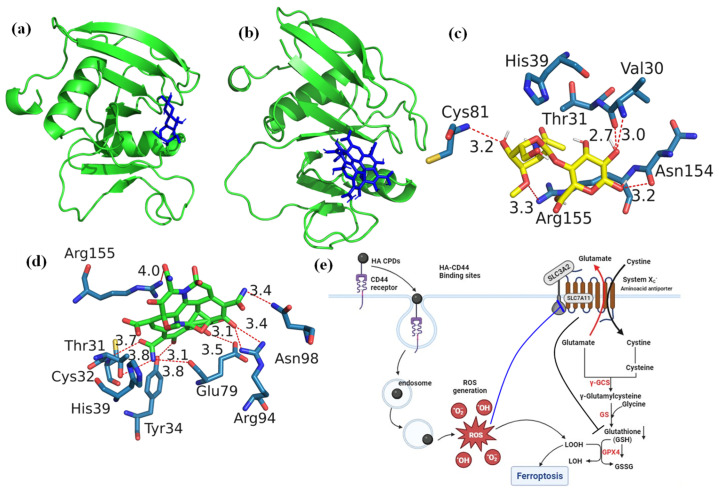
Docking analysis of (**a**) HA, (**b**) HA-CQDs, demonstrating their interaction with the 3D structure of the CD44 receptor, and identifying the best-docked sites of (**c**) HA, (**d**) HA-CQDs in the CD44 receptor, and (**e**) HA-CQDs based on the Ferroptosis anticancer mechanism.

Molecular docking studies are crucial in drug discovery and development, particularly in experimental drug delivery [30]. These studies help identify potential targets by predicting interactions between ligand molecules and specific biological targets. This predictive capability is essential for identifying lead ligand molecules with high binding affinities and examining how changes to the chemical ligand structure may affect binding affinity and interactions with the target [30]. Moreover, molecular docking provides valuable insights and predictions that inform the selection, optimization, and understanding of drug candidates. Ultimately, this increases the efficiency and success rate of experimental drug delivery efforts. CD44, a type I transmembrane receptor protein, is expressed in various human cell types, including leukocytes, endothelial cells, and fibroblasts, and can associate with the HA ligand. HA, an anionic glycosaminoglycan, is increasingly recognized as a promising ligand for targeting CD44 receptors [31]. These receptors are overexpressed in several solid tumors and are implicated in cancer metastasis [32]. Treatment of triple-negative breast cancers (TNBCs) is notably challenging, given its aggressive spread, slow recovery rate, high metastatic potential, and rapid relapse compared to other breast cancer subtypes [33]. Current therapies show limited effectiveness against TNBCs. Consequently, enhancing anticancer activity while minimizing adverse effects requires targeted drug delivery to the cancer cells. Targeting CD44-expressing TNBCs by binding them to the CD44 receptor can serve as a foundational strategy for numerous treatments [34]. The CD44 receptor-targeted HA-based nano platform has shown improved therapeutic efficacy in treating TNBCs. In this study, the docking of HA and HA-CQDs with CD44 is illustrated in Figure 5a,b. The binding affinity of HA and HA-CQDs to the CD44 receptor [35] was determined to be −6.5 and −7.0 kcal mol^−1^, respectively. The HA-CQD ligands exhibited higher affinity values than the HA ligands, thereby enhancing the binding affinity of HA-CQDs toward the CD44 receptors. This was confirmed by using two distinct cancer cell lines, MDA-MB-231 (CD44 positive) and HePG2 (CD44 negative), in cytotoxicity assays. TNBCs demonstrated greater anticancer activity than HepG2, indicating CD44-mediated targeted delivery for HA-CQDs.

Figure 5c,d depict the optimal accommodation of HA and HA-CQDs to CD44 using blind docking. As illustrated in Figure 5c, HA forms five conventional hydrogen bonds (weaker interactions between the oxygen atoms due to electrostatic attraction between a proton in one molecule and an electronegative donor atom, such as a polarized carbon atom) with VAL30A, CYS81A, ASN154A, and ARG155A of the CD44 receptor. Two hydrophobic interactions occur between water and hydrophobic molecules associated with THR31A and ARG155A of the CD44 receptor, binding with the HA ligand. Additionally, HA establishes two salt bridges with its carboxylate group as an intrinsic ligand, creating interactions with HIS39A and ARG155A of the CD44 receptor.

The HA-CQD-based docking model in Figure 5d shows all interactions between the HA-CQD ligand and the CD44 receptor. The docking results demonstrate that the HA-CQD ligands can form hydrophobic interaction bonds with THR31A of the CD44 receptor. There are nine conventional hydrogen bonds between the oxygen and nitrogen atoms bonding with HA-CQD ligands and THR31A, CYS32A, TYR34A, GLU79A, ARG94A, ASN98A, and ARG155A of the CD44 receptor. Furthermore, two carboxylate groups of HA-CQD ligands form salt bridges with the amino acids HIS39A and ARG155A of the CD44 receptor.

### 3.6. Biological Significance of HA System and HA-CD44 Binding Affinity

HA is a remarkable glycosaminoglycan found in the extracellular matrix of various tissues [36]. Understanding its structure, functions, and interactions is crucial for comprehending its impact on cellular processes and its significance in health and disease. HA is a linear polysaccharide, comprising long-chain units of N-acetyl-β-D-glucosamine and β-D-glucuronate residues [37]. These residues are linked at the 1–3 and 1–4 positions, forming a lengthy, unbranched chain of disaccharide units. This unique structure contributes to HA’s exceptional water-holding capacity, which is essential for maintaining tissue hydration and structural integrity. HA molecules are abundantly present in the extracellular matrix (ECM), particularly in connective tissues such as skin and cartilage, aiding in the maintenance of tissue architecture and hydration. Furthermore, HA plays a pivotal role in various cellular processes, including cell proliferation (affecting cell cycle progression and growth), adhesion (facilitating cell interactions with the ECM and adjacent cells), morphogenesis (influencing tissue morphogenesis and the development of numerous organs and structures), differentiation (of various cell types, including stem cells), and inflammation (stimulating inflammation through interactions with specific receptors). HA’s effects are mediated through specific cell surface receptors, with the primary receptors being CD44 and the receptor for HA-mediated motility (RHAMM) [38,39]. CD44 is a complex glycoprotein, while RHAMM is a soluble receptor [40]. These receptors bind and transduce HA-mediated signals, influencing various cellular responses. With regard to the role of HA and its interaction with CD44 in tumor progression [27], CD44 is often overexpressed and activated in solid tumors, leading to the CD44 receptor’s strong affinity for binding and internalizing HA nanoplatforms.

CQDs offer the potential for developing theragnostic nanoparticles, combining therapeutic and diagnostic capabilities for cancer imaging and therapy. The indiscriminate distribution of CQDs in healthy tissues can lead to unforeseen biotoxicity, significantly limiting the potential for advancing diagnostic and therapeutic applications [41] of carbon dot nanostructures. Addressing the challenges of targeting CQDs to tumor sites, recent decades have seen the synthesis of CQDs using various raw materials such as HA, chitosan, and sodium alginate. Among these, HA stands out as a carbon source precursor, primarily because it utilizes compounds with targeted specificity as precursors for CQD synthesis [14]. This distinctive approach imparts a self-targeting function to CQDs. HA’s ubiquity as a glycosaminoglycan in mammalian tissues and the extracellular matrix further enhances its significance. Notably, HA ligands interact with CD44 receptors which are positively expressed and activated in cancer cells [14]. This heightened affinity for CD44 receptors makes HA an effective targeting molecule for cancer cells, offering remarkable specificity in contrast to normal cells. HA provides a viable approach for producing self-targeted CQDs for cancer drug delivery. These properties significantly improve the use of HA in the clinical industries for cancer diagnosis. Importantly, CQDs possess various functional groups on the surface, such as -NHCO-, C=O, C-O-C, C-H, OH-, and COO- groups [14], contributing to their excellent quantum yield and biocidal properties. The sp2 carbon structures in the CQDs’ and HA-CQDs’ surface site carboxyl groups (COO-) can act as electron donors, facilitating efficient charge transfer to H_2_O_2_, resulting in the formation of a •O_2_^−^ radical and a proton H^+^:

HA-CQDs + H_2_O_2_ → HA-CQDs •O_2_^−^ + H^+^

The superoxide radical reacts with another molecule of H_2_O_2_, forming the hydroperoxyl radical (•OOH) and a hydroxide ion (OH^−^):

HA-CQDs •O_2_^−^ + H_2_O_2_ → HA-CQDs + •OOH^−^ + OH^−^

The hydroperoxyl radical decomposes to form a hydroxyl radical (•OH) and a hydroperoxide ion (OOH^−^):

•OOH^−^ → •OH + OOH^−^

The hydroxyl radicals generated from the reaction can cause oxidative damage to cells. Furthermore, ferroptosis in CQDs is primarily contingent upon the interplay of multiple factors, including iron dependency, lipid peroxidation, their relatively small size, increasing surface area, and surface chemistry parameters are inducted into the ferroptosis effects. The CQDs generate intercellular ROS in the cytoplasm, especially for GSH oxidase, resulting in subsequent oxidative stress that can damage the biological systems and lead to cancer cell death. Especially ferroptosis, a new type of programmed cell death, which is of great interest in cancer therapy. Systematically, ferroptosis is predominantly downregulated by the GSH redox system [42]. Biochemically, biological systems undergoing lipid peroxidation-based ferroptosis exhibit harmful peroxidation of PUFAs into PL-PUFAs, increasing the intracellular ROS levels [43]. The enzyme GPX4 is pivotal in converting lipid peroxides (L-OOHs) into their corresponding lipid alcohols [44]. GPX4’s activity is directly contingent on GSH availability, which can be synthesized from cysteine and glutamate [45].

Ferroptosis is triggered by lipid hydroperoxides in cellular membranes. GPX4, a crucial enzyme within the glutathione antioxidant system, uses GSH as a substrate to convert L-OOH into non-toxic L-OH, thus preventing lipid peroxidation due to increased intracellular ROS [44]. The biosynthesis of the GSH substrate is tightly regulated by system Xc- [46]. SLC7A11 mediates the cystine/glutamate exchange performed by the system Xc- unit (Figure 5e). This cystine transporter, referred to as SLC7A11, is critical for cellular viability as deprivation of cystine leads to significant cell death. However, blocking the activity of SLC7A11 effectively inhibits cystine uptake, causing insufficient GSH conversion and allowing lipid peroxidation products to accumulate in the cytoplasm, leading to ferroptosis. Blocking SLC7A11, depleting GSH, and inhibiting GPX4 can all disrupt intracellular redox balance, diminish cell antioxidant capacity, and release the restraint on lipid peroxides, resulting in lipid peroxidation-induced ferroptosis [47].

### 3.7. In Vitro Cytotoxicity

**Figure 6:**
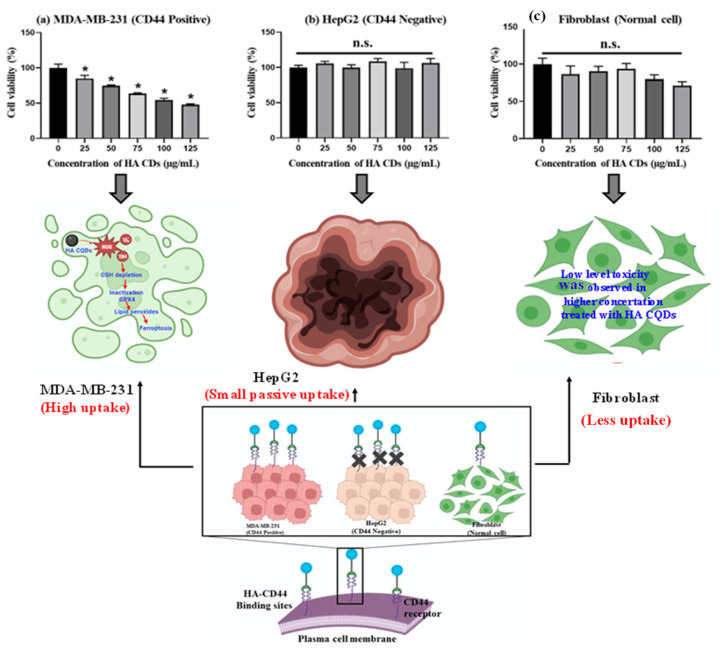
In vitro anticancer effect of HA-CQDs. Cell viability of (**a**) MDA-MB-231 (**b**) HepG2, and (**c**) Fibroblast cells when treated with various concentrations of HA-CQDs for 24 h using WST-1 assay. (*: statistical difference compared to 0 µg/mL, n.s.: non-statistical differences).

TNBCs, accounting for 15–20% of all breast cancers, are the most aggressive type, and the lack of effective diagnostics along with metastasis is the primary cause of morbidity and mortality in TNBC patients [48]. TNBCs predominantly express the CD44 receptor-positive cancer cells [49], which we examined to assess their tumor-targeting capability in delivering HA-CQD systems incorporating ligands for the CD44 receptor model, owing to HA’s active targeting of TNBCs that overexpress the CD44 receptor. We evaluated the cytotoxicity of HA-CQDs in MDA-MB-231 (CD44 positive), HepG2 (CD44 negative), and fibroblast as shown in (Figure 6a–c) at various concentrations (0–125 μg/mL) (comparative studies given in Table 1). In our results, HA-CQDs demonstrated superior anticancer effects in MDA-MB-231 possessing a positive CD44 receptor compared to HepG2, which is negative for CD44. The anticancer effects rely on surface-active ligands for tumor recognition, selective binding to cancer cells, cellular uptake (CD44-mediated endocytosis) [31,50], and the concentration of CQDs. HA-CQDs selectively bind to TNBCs but not to HepG2 cells. Since TNBCs overexpress CD44 surface ligands, unlike HepG2 cells which lack these ligands, resulting in limited passive cellular uptake, no significant anticancer effects were observed, similar to the control group. In the case of fibroblasts, they maintained cell viability at concentrations ranging from 0 to 125 μg/mL (Figure 6c).

In addition, HA-CQD physio-chemical properties contribute to their anticancer activity through features such as small particle size, increased surface area sp2 carbon structures with delocalized π electron donors in the CQDs [51], surface chemistry (carbonyl, hydroxy, amine, and carboxyl groups), and the induction of ferroptosis. Guangzhe Zheng et al. reported that ferroptosis influenced the size of silver NPs particles specifically at 10 nm and 50 nm. The 10 nm Ag NPs exhibited more potent ferroptosis-based anticancer effects than the 50 nm Ag NPs [52]. Tian et al. (2022) demonstrated that 5 nm small-size particles can accumulate in the nucleus, and small particle size effectively triggered the ROS compared to larger particles, attributed to the release of Fe^2+^-induced ferroptosis-based anticancer activity [53]. In this work, the size of HA-CQDs is 2.2 nm, with a surface area of 35.736 m^2^/g. The smaller size and higher surface area of HA-CQDs lead to increased ROS generation upon contact with cancer cells, causing GSH depletion and lipid peroxidation, resulting in substantial anticancer effects. In contrast, MDA-MB-231 (CD44 positive) showed anticancer activity, whereas HepG2-treated cells did not exhibit any anticancer effects because CD44 expression was negative. Fibroblast cells, which showed normal CD44 expressions, exhibited a noticeable decline in HA-CD44 binding expression. Furthermore, fibroblasts demonstrated an IC50 concentration of 125 μg/mL, resulting in approximately 90 ± 5% cell viability with statistical significance (*p* < 0.05). Notably, HA-CQDs have been categorized as biocompatible materials characterized by minimal cytotoxicity. The outcomes from experiments involving HA-CQDs indicate their potential for safe integration into injectable formulations designed to deliver CD44-targeted anticancer agents, suggesting that HA-CQDs hold promise as a viable platform for advancing targeted anticancer therapies, particularly given their compatibility and minimal impact on cellular viability.

Cell death is an inevitable and essential aspect of life in both physiological and pathological conditions, marking the end of a cell’s existence [54]. Broadly, cell death can be categorized into apoptosis and necrosis. In recent decades, additional distinct biological processes and pathophysiological characteristics, such as autophagy and necrotic apoptosis, have emerged alongside necrosis and apoptosis [55]. Ferroptosis is a crucial form of oxidative stress-dependent cell death and has gained significant attention in clinical industries [56]. Ferroptosis occurs through various mechanisms including iron-dependence, lipid peroxidation, GSH depletion, and GPX4 inactivation [57]. Ferroptosis is a non-apoptotic cell death mode, characterized by lipid ROS generation. Morphologically, ferroptosis is distinct from necrosis, apoptosis, and autophagy [58]. It lacks the typical features of necrosis, such as cytoplasmic swelling, organelle enlargement, and cell membrane rupture.

For the live and dead study, MDA-MB-231, HepG2, and fibroblast cells were exposed to HACQDs at concentrations ranging from 0 to 100 μg/mL as shown in Figure S4a–c. Figure S4a shows control cells displaying a uniform green color. Control cells exhibited an intact cell membrane and uniform structure. After 24 h of treatment with 100 μg/mL HA-CQDs, MDA-MB-231 cells showed fewer live cells (depicted in green) than the control group, indicative of ferroptosis-based cell death (a non-apoptotic mode of cell death) [58]. However, results for HepG2 cells treated with HA-CQDs at 100 μg/mL concentrations revealed no significant anticancer difference in the live cells compared to untreated (control) cells due to lower uptake of HA-CQDs in HepG2 cells (CD44 negative) (Figure S4c). When exposed to HA-CQDs at 100 μg/mL concentrations, fibroblast cells (with low CD44 binding sites) maintained a uniform fusiform structure and 90 ± 5% cell viability. HA-CQDs demonstrated potential for use in advanced biomedical industries, especially in CD44 receptor-mediated ferroptosis-based targeted drug delivery applications.

### 3.8. Detection of Intercellular ROS

**Figure 7:**
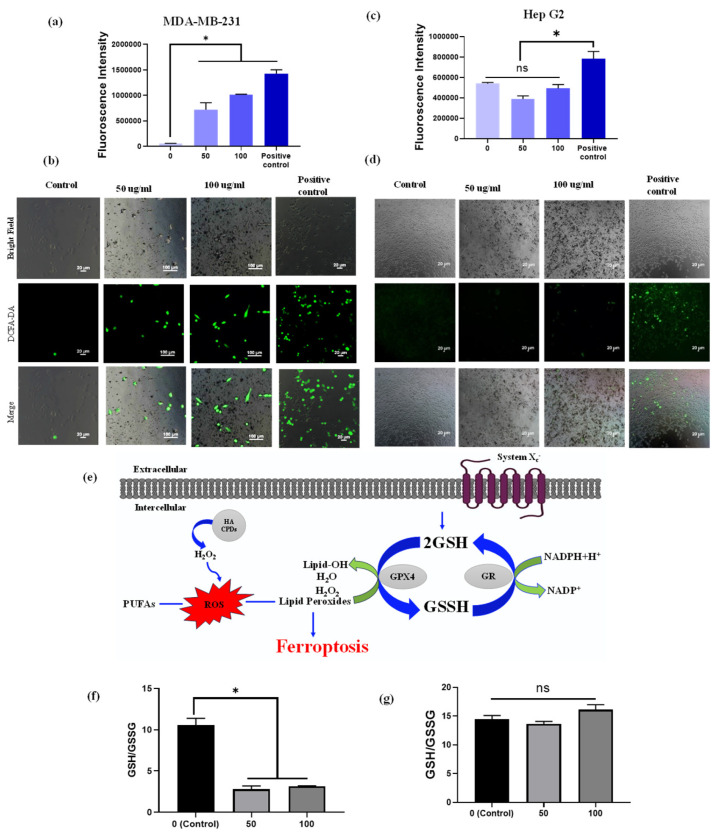
Intracellular ROS measurement. ROS generation was determined using the DCFH-DA probe. Fluorescence intensity and fluorescence images of DCFH-DA after exposure to HA-CQDs (0, 50, and 100 μg/mL) for 6 h in MDA-MB-231 cells (**a**,**b**) and (**c**,**d**) HepG2 cells. (II) The ratio of GSH/GSSG includes (**e**) the mechanism of GSH depletion, (**f**) the ratio in MDA-MB-231 cells, and (**g**) the ratio in HepG2 cells treated with 0, 50, and 100 μg/mL of HA-CQDs for 24 h. (*: statistical difference compared to 0 µg/mL, n.s.: non-statistical differences).

To investigate the potential of HA-CQDs at different concentrations (0–100 µg/mL) and a positive control (H_2_O_2_) for generating intracellular ROS, we employed the 2’,7’-dichlorodihydrofluorescein diacetate (DCFH-DA) (Sigma-Aldrich, St. Louis, MO, USA) probe, which specifically indicates intracellular ROS generation [59]. DCFH-DA, in its diacetate form, is used to detect ROS within cells due to its ability to permeate cell membranes. Once inside, intracellular esterases cleave the two acetate groups from DCFH-DA. Moreover, peroxidases play a vital role in oxidizing DCFH by H_2_O_2_. It is important to note that compounds such as hematin or cytochrome c can oxidize DCFH, enhancing the probe’s fluorescence, even without H_2_O_2_ production [60]. When subjected to light radiation (UV or visible light), DCFH can undergo photo reduction, as shown in Figure S5. The resultant fluorescent product emits green fluorescence at 535 nm upon excitation at 485 nm. The oxidation of the probe generates a semiquinone radical (DCF•−), which reacts with O_2_ to form •O_2_^−^. The dismutation of •O_2_^−^ leads to the production of H_2_O_2_, further promoting the oxidation of DCFH. This oxidation ultimately results in the formation of a fluorescent product, dichlorofluorescein (DCF), which exhibits strong fluorescence in the presence of ROS [61]. The untreated HA-CQD control cells showed no fluorescence intensity in this study. CD44 overexpressing MDA-MB-231 cells and CD44 negative HepG2 cells treated with a range of HA-CQD concentrations (0 to 100 µg/mL), including the positive control (H_2_O_2_), demonstrated strong DCF fluorescence. Notably, the positive control H_2_O_2_ showed the highest fluorescence intensity. In the case of HA-CQDs treated with MDA-MB-231 cells, the fluorescence intensifies as the concentration of HA-CQDs increases (Figure 7a), indicating enhanced ROS generation in a concentration-dependent manner. Similarly, HA-CQDs treated with HepG2 exhibited no fluorescence intensity at lower and higher concentrations (Figure 7b), and the positive control H_2_O_2_-treated group exhibited strong fluorescence intensity, suggesting that HepG2 cells showed low CD44 expression due to the absence of CD44 receptor-mediated endocytosis for HA-CQDs.

From the fluorescence microscopic image analysis of MDA-MB-231 cells treated with concentrations of 50 and 100 µg/mL, the untreated (control) group displayed 1% green fluorescence, indicating ROS production potentially due to external factors such as UV radiation exposure. The HA-CQDs treated MDA-MB-231 groups showed strong green fluorescence of DCF. As the concentration of CQDs increased, a higher generation of ROS was observed in the MDA-MB-231 cells (green fluorescence cells) because HA-CQDs exhibit concentration-dependent anticancer effects in MDA-MB-231 (CD44 positive) through internalization via CD44 receptor-mediated endocytosis. Once inside the cytoplasm, the sp2-bonded HA-CQD structures use their delocalized π electron donors and the COO- groups on their surface chemistry as electron donors for efficient charge transfer to hydrogen peroxide (H_2_O_2_). This interaction results in the formation of •O_2_^−^ and •OH radicals. Consequently, strong DCF fluorescence binds with ROS, resulting in the emission of green fluorescence (Figure 7a). The H_2_O_2_-treated positive control group also displayed strong green fluorescence DCF, similar to the HA-CQD treated group. However, in the HepG2-treated group, the control cells showed no green fluorescence. At both lower and higher concentrations (50 and 100 µg/mL), the HA-CQD treated groups exhibited 2–5% green fluorescence of DCF, which may be due to some CQD uptake via passive uptake mediated ROS generation.

### 3.9. GSH Depletion

The GSH/glutathione disulfide (GSSG) system is essential for maintaining cellular redox balance [62] (Figure 7a). Depletion of intracellular GSH concentration occurs when GPX4 becomes inactive, resulting in the accumulation of toxic lipid reactive oxygen species (L-ROS). This accumulation leads to lipid peroxidation and contributes to ferroptosis-dependent anticancer activity. Our study examined GSH depletion in MDA-MB-231 and HepG2 cells following treatment with HA-CQDs at concentrations of 0, 50, and 100 µg/mL for 24 h. As indicated in Figure 7b, the ratio of GSH/GSSG substantially decreased in the HA-CQD-treated MDA-MB-231 group compared to the control group. The decline in GSH may be attributed to its oxidation into GSSG by GSH oxidase activity, which promotes ferroptosis-based cancer cell death by reducing GPX4-catalyzed lipid damage. However, no significant difference was observed in the GSH/GSSG ratio in the HA-CQD-treated HepG2 group (Figure 7c).

### 3.10. mRNA Expression Profiles of Ferroptosis Markers and Lipid Peroxidation (MDA Assay)

**Figure 8:**
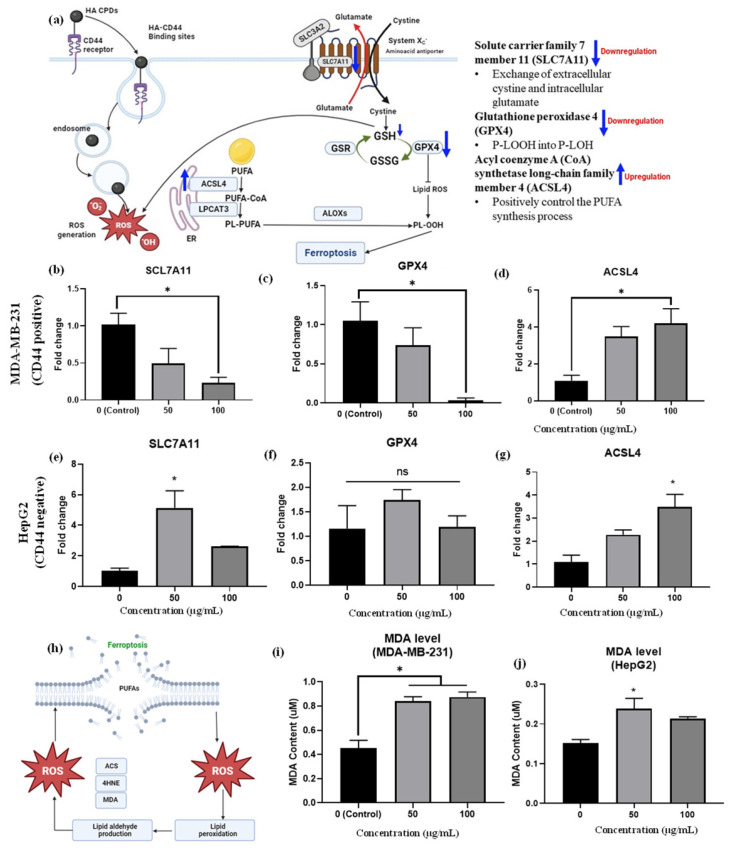
(**a**) Provides insights into mRNA expression of ferroptosis-related genes. The mRNA expressions of (**b**) SLC7A11, (**c**) GPX4, and (**d**) ACSL4 were measured in MDA-MB-231 cells treated with HA-CQDs; and expressions of (**e**) SLC7A11, (**f**) GPX4, (**g**) ACSL4 were evaluated in HepG2 cells treated with HA-CQDs. GAPDH was utilized as a housekeeping gene. * indicates a significant difference with the control (*p* < 0.05). A ferroptosis mechanism based on lipid peroxidation featured (**h**) MDA levels in (**i**) MDA-MB-231 group and (**j**) HepG2 group after treatment with HA-CQDs at concentrations of 50 and 100 μg/mL for 24 h.

The mRNA expression of ferroptosis-related genes in MDA-MB-231 triple-negative breast cells and HepG2 liver cancer cells was assessed after treatment with HA-CQDs at concentrations of 50 and 100 μg/mL, exploring the anticancer activity related to ferroptosis (see Figure 8b–d). Our qRT-PCR analysis confirmed that these gene expressions, specifically SLC7A11, ACSL4, and GPX4 [9], might serve as early indicators of ferroptosis in HA-CQD-treated MDA-MB-231 and HepG2 cells.

SLC7A11 is a multi-pass transmembrane protein capable of cystine uptake via the system Xc- amino acid antiporter. However, low gene expression levels of SLC7A11 suggest a limited rate of cysteine precursor supply for GSH synthesis, as intracellular cystine is primarily taken up by the SLC7A11 protein subunit (Figure 8a). Studies show that ZnO (10 μg/mL) nanoparticles induce ferroptosis in cancer cells, evidenced by decreased SLC7A11 mRNA expression levels compared to the control group [9]. Additionally, 100 μg/mL nitrogen-doped graphene quantum dots inhibited SLC7A11 protein expression levels [63], indicating induced ferroptosis effects. In this study, HA-CQDS were administered to both CD44-positive TNBCs and CD44-negative HepG2 liver cancer cells. The TNBCs exhibited a high uptake of HA-CQDs owing to the downregulation of SLC7A11 mRNA expression, as evidenced by the comparison with the control group (Figure 8b). Conversely, the HepG2 group showed up-regulation of SLC7A11 mRNA levels compared to the control (Figure 8e), due to a lower uptake by CD44-negative cells, and HepG2 cells treated with HA-CQDs did not experience GSH depletion (Figure 7c) since cysteine was adequately supplied through uptake by the system Xc- subunit SLC7A11, which conferred ferroptosis resistance [64].

Furthermore, ferroptosis can be triggered by extrinsic pathways that suppress the SLC7A11 protein system, responsible for the exchange of extracellular cystine and intracellular glutamate. SLC7A11 plays a vital role in maintaining the GSH antioxidant system by activating the GPX4 enzyme, which catalyzes the conversion of L-OOH to L-OH, a critical regulatory step in ferroptosis [65]. Downstream of SLC7A11, GPX4 is crucial in ROS accumulation from lipid peroxidation, reducing the primary antioxidant GSH to GSSG and thereby inactivating GPX4 [66]; these results confirm it as a biomarker of ferroptosis (Figure 8a).

Indeed, in the MDA-MB-231 group treated with HA-CQDs, GPX4 mRNA levels were downregulated. Furthermore, as the concentration of 100 μg/mL of HA-CQDs increases, a higher level of downregulation in GPX4 expression is observed compared to the control group (Figure 8c). HA-CQDs generate ROS, due to GSH depletion and GPX4 enzyme inactivation, which contributes to lipid peroxidation and ferroptosis, as indicated by GPX4 levels [38]. Higher GPX4 expression was observed in HePG2 cancer cells (Figure 8f), indicating that anticancer effects do not occur via ferroptosis because of the lower uptake of HepG2 cells (CD44 negative).

ACSL4 protein positively regulates the synthesis of PUFAs [67]. The ACSL4 protein also contributes to the buildup of lipid intermediates, which are compounds involved in lipid metabolism [68] (Figure 8a). This lipid accumulation facilitates ferroptosis. Conversely, higher gene expression levels of ACSL4 can increase cellular susceptibility to ferroptosis, a type of cell death caused by lipid peroxide accumulation [42]. In our results, increasing concentrations of HA-CQD treatment led the MDA-MB-231 group to exhibit an upregulation of ACSL4 mRNA levels compared to the control group (Figure 8d). On the contrary, an increase in ACSL4 mRNA levels was seen in the HepG2 group (Figure 8f). For TNBCs, ACSL4 expression levels are two-fold higher than those in HepG2. HepG2 cells with ACSL4 gene expression may experience external stress, leading to passive HA CQD uptake and subsequent ROS generation.

As the concentration of HA-CQDs increases, the MDA-MB-231 group exhibits a downregulation in SLC7A11 and GPX4 mRNA levels and an upregulation in ACSL4 mRNA levels compared to the control group. These results suggest that HA-CQDs selectively bind to the CD44 receptor, mediating ferroptosis-dependent cancer cell death. The HA-CQD nanoplatform could potentially induce ferroptosis in MDA-MB-231 cells.

Malondialdehyde (MDA) is an aldehyde formed as a secondary product during lipid peroxidation. Extensively referenced in the literature, MDA serves as a widely employed, convenient biomarker for assessing lipid peroxidation [9]. As illustrated in Figure 8h, increased lipid peroxidation in cancer cells is confirmed using a malondialdehyde (MDA) assay. In this study, the increase in MDA levels was three-fold higher in the MDA-MB-231 group than in the HepG2 group (Figure 8i–j).

**Table 1:** The comparison studies of CD44-targeted drug delivery with HA system.

S. No	Molecules	Target	Cancer Cells	Efficacy
1	CQD–HA–PEI@GNR–DOX	CD44	Breast cancer cells (MCF7) [69]	200 μg mL^−1^
2	Verapamil-loaded hyaluronic acid-modified carbon quantum dots	CD44	SH-Sy5y human cell line Neuro 2a mouse neuroblastoma cell line [70]	3.75 μg/mL
3	Graphene quantum dot-hyaluronic acid nanocomposites	CD44	Breast cancer (MCF-7) [71]	500 µg/mL
4	HA CQDs	CD44	Cervical cancer cells [72]	100 μg/mL
5	HA-CQD@p-CBA-DOX	CD44	Breast cancer cell line [73]	90%

For the potential off-target effects of HA–CQDs, we conducted a detailed investigation of the specific binding affinity through CD44-mediated endocytosis using three different cell types. A normal fibroblast cell line (low CD44 expression) was included to assess non-specific interactions and potential off-target effects. Additionally, two cancer cell lines were analyzed: **MDA-MB-231** (triple-negative breast cancer, CD44-positive) and **HepG2** (liver cancer, CD44-negative). The inclusion of **MDA-MB-231 cells** was critical for examining selective targeting, while **HepG2 cells** served as a control to evaluate specificity. This analysis confirmed that HA–CQDs selectively interact with CD44-positive cancer cells, minimizing non-specific interactions with normal or CD44-negative cells, which is crucial for reducing off-target effects.

Regarding the challenges in large-scale synthesis and formulation, we have discussed several factors in the revised manuscript. Scaling up production from laboratory to industrial levels can result in inconsistencies in **size**, **quality**, **and purity**, which may affect the effectiveness of HA–CQDs. Additionally, **cost efficiency**, **long-term stability**, and **bioavailability** are critical considerations for clinical use. The purification process needs to ensure biocompatibility, and regulatory approval requires meeting stringent safety and efficacy standards. Addressing these challenges is vital for the successful clinical translation of HA–CQDs and their widespread use.

## 4. Conclusions

We developed a CD44-targeted HA-CQD nanoplatform synthesis through a one-pot hydrothermal treatment. The intrinsic properties of the developed HA-CQDs include excellent water-dispersibility, biocompatibility, and CD44 receptor-mediated endocytic cellular internalization, promoting ferroptosis-based programmed cell death. Our method employs HA-CQD-based biomaterials that enable cancer cells to recognize CD44 ligands on the surface of triple-negative breast cancer cells. Following HA-CQD cellular internalization via CD44-mediated endocytosis, the release of HA-CQDs in endosomes generates increased amount of ROS, and excessive ROS leads to lipid peroxidation, triggering ferroptosis via downregulation of GSH and SLC7A11. We further found that HA and HA-CQDs interact more effectively with CD44 ligands using an in-silico method (docking analysis). HA-CQDs exhibit more interactions with CD44 ligands compared to HA alone. These results suggest that customized ferroptosis cancer therapies could be effective for treating a variety of solid tumors.

## Data Availability

The original contributions presented in this study are included in the article. Further inquiries can be directed to the first author.

## Supplementary Materials

The following supporting information can be downloaded at https://www.mdpi.com/article/10.3390/ma18092139/s1, Figure S1: Lipid peroxidation generation of HA CQDs (MDA assay); Figure S2: Gaussian decomposition FTIR spectrum of (a1,a2) hyaluronic acid and (b1,b2) HA CQDs; Figure S3: (a) UV–Visible absorption spectra of HA CQDs, (b) color emission for UV–Visible with NIR region and (c,d) PL spectra of HA CQDs; Figure S4: In vitro anticancer effect of HA CQDs. Fluorescence images of cell viability in (a) MDA-MB-231, (b) HepG2, and (c) Fibroblast after treating with 100 µg/mL of HA CQDs for 24 h using live and dead assay; Figure S5: DCFH-DA intercellular ROS mechanism for HA CQDs; Table S1: CD44 (4MRD) protein amino acid position parameters; Table S2: Primer sequence for RT-PCR; Table S3: Vibrational assignment of hyaluronic acid and HA CQDs.
