# Supplementary material for: CD44 Receptor-Mediated Ferroptosis Induction by Hyaluronic Acid Carbon Quantum Dots in Triple-Negative Breast Cancer Cells Through Downregulation of SLC7A11 Pathway
Source: Materials (Basel). 2025 May 6;18(9):2139. doi: 10.3390/ma18092139 (PMC12073876; doi:10.3390/ma18092139)
Supplement: Supplementary file 1 [file materials-18-02139-s001.zip › materials-3594539-supplementary.pdf]

Supplementary Materials

CD44 Receptor-Mediated Ferroptosis Induction by Hyaluronic Acid Carbon Quantum Dots in Triple-Negative Breast Cancer Cells through Downregulation of the SLC7A11 Pathway

Karthikeyan Chandrasekaran[1,#], Chae Eun Lee[1,#], Seojeong Yun[1], Ashok Kumar Jangid[1], Sungjun Kim[1], and Kyobum Kim[1*]

[1]Department of Chemical & Biochemical Engineering, Dongguk University, Seoul, 04620, Republic of Korea

#These authors contributed equally to this work

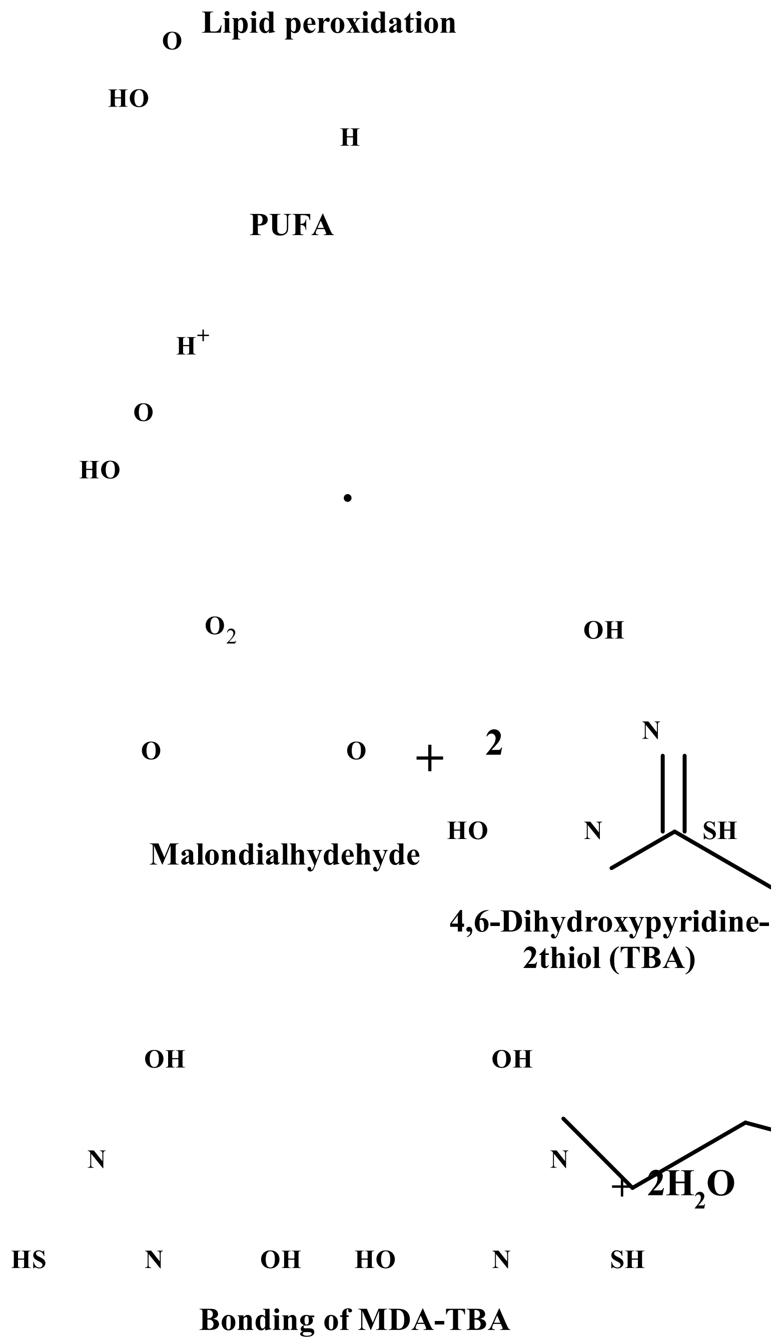

Figure S1. Lipid peroxidation generation of HA CQDs (MDA assay).

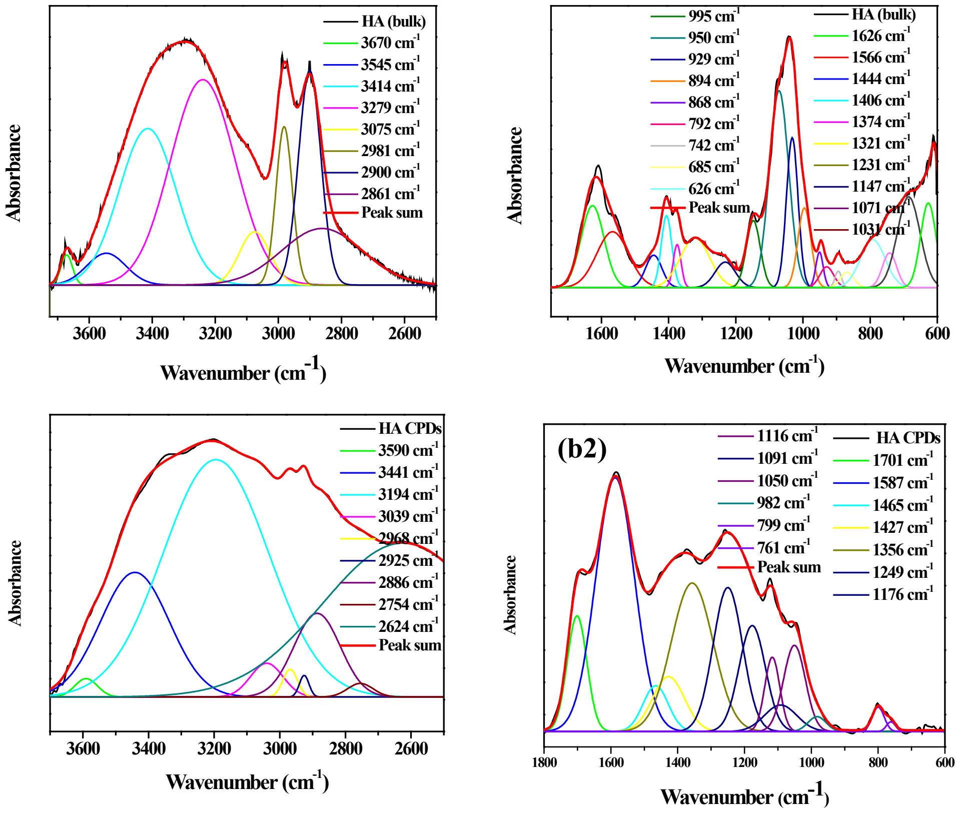

Figure S2. Gaussian decomposition FTIR spectrum of (a1-a2) Hyaluronic acid and (b1-b2) HA CQDs.

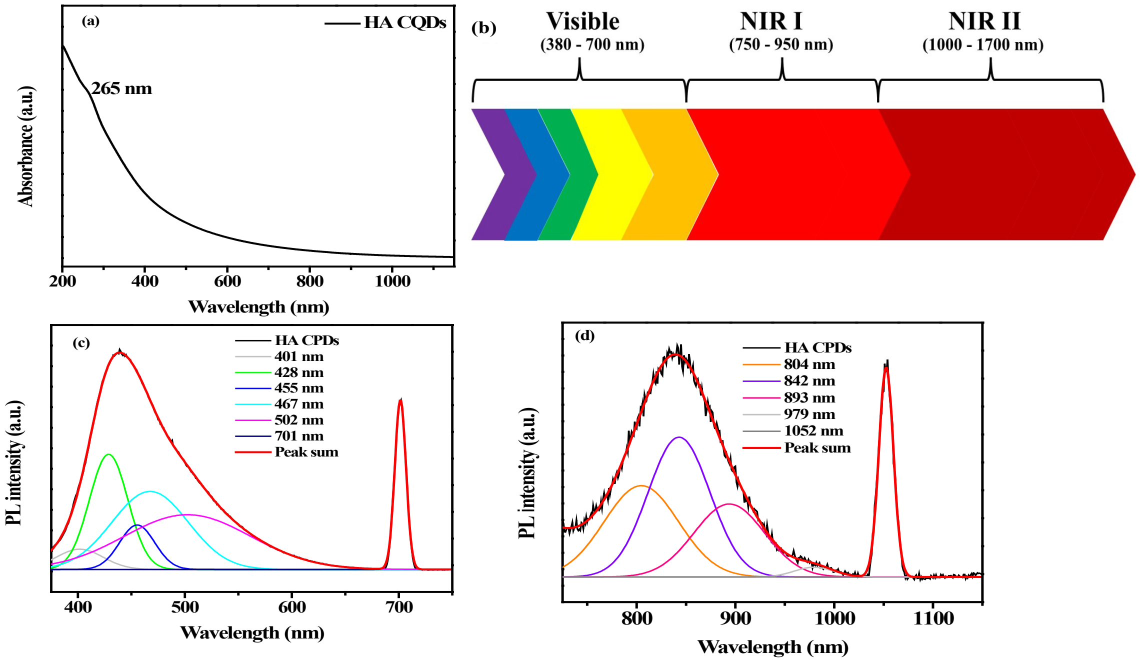

Figure S3. (a) UV-Visible absorption spectra of HA CQDs, (b) color emission for UV-Visible with NIR region and (c-d) PL spectra of HA CQDs

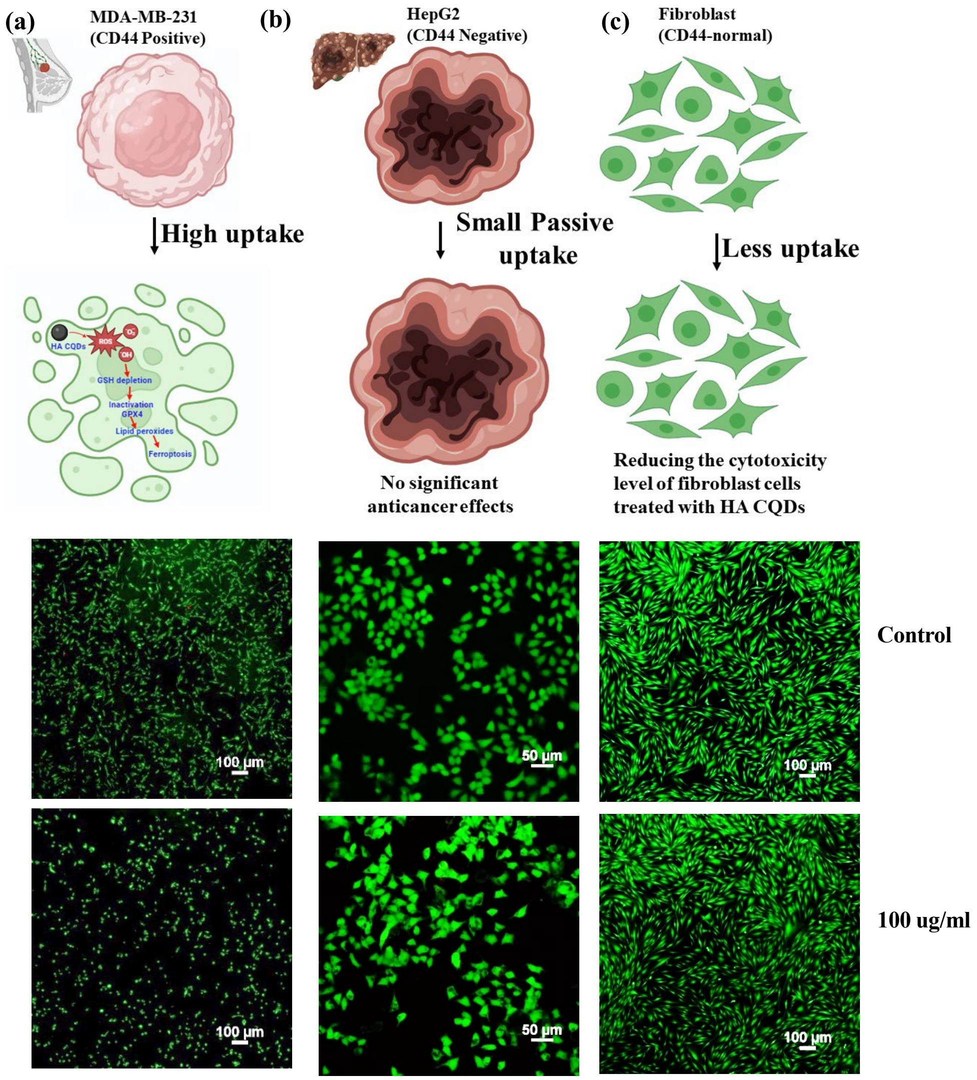

Figure S4. In vitro anticancer effect of HA CQDs. Fluorescence images of cell viability in (a) MDA-MB-231 and (b) HepG2 and (c) Fibroblast after treating concentrations 100 µg/mL of HA CQDs for 24 h using live and dead assay.

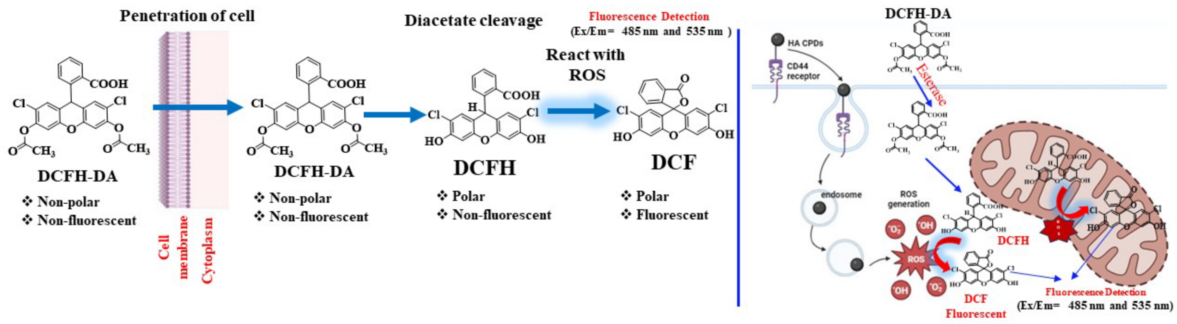

Figure S5. DCFH-DA intercellular ROS mechanism for HA CQDs.

Table S1. CD44 (4MRD) protein amino acid position parameters.

PocID	Chain	SeqID	AA	Atom
1	A	29	ASN	C
1	A	29	ASN	O
1	A	29	ASN	CB
1	A	30	VAL	N
1	A	30	VAL	CA
1	A	30	VAL	C
1	A	30	VAL	O
1	A	31	THR	CA
1	A	31	THR	CB
1	A	31	THR	CG2
1	A	32	CYS	N
1	A	32	CYS	CB
1	A	32	CYS	SG
1	A	34	TYR	CE2
1	A	34	TYR	CZ
1	A	34	TYR	OH
1	A	39	HIS	ND1
1	A	39	HIS	CE1
1	A	41	GLU	CB
1	A	41	GLU	CD
1	A	41	GLU	OE1
1	A	41	GLU	OE2
1	A	77	GLY	O
1	A	78	PHE	CA
1	A	78	PHE	CD1
1	A	78	PHE	CE1
1	A	79	GLU	N
1	A	79	GLU	O
1	A	80	THR	CA
1	A	80	THR	CB
1	A	80	THR	CG2
1	A	81	CYS	N
1	A	82	ARG	CG
1	A	82	ARG	NE
1	A	82	ARG	CZ
1	A	82	ARG	NH1
1	A	82	ARG	NH2
1	A	153	VAL	O
1	A	154	ASN	C
1	A	155	ARG	N
1	A	155	ARG	CA
1	A	155	ARG	CB
1	A	155	ARG	CG
1	A	155	ARG	CD
1	A	155	ARG	NE
1	A	155	ARG	CZ
1	A	155	ARG	NH1
1	A	155	ARG	NH2

Table S2. Primer sequence for RT-PCR

Protein	Forward	Reverse
GAPDH	GGG AGC CAA AAG GGT CAT CA	TGA TGG CAT GGA CTG TGG TC
GPX4	GAG GCA AGA CCG AAG TAA ACT AC	CCG AAC TGG TTA CAC GGG AA
SLC7A11	TCT CCA AAG GAG GTT ACC TGC	AGA CTC CCC TCA GTA AAG TGA C
ACSL4	ACT GGC CGA CCT AAG GGA G	GCC AAA GGC AAG TAG CCA ATA

Table S3. Vibrational assignment of Hyaluronic acid and HA CQDs.

S. No.	Hyaluronic acid (cm^{-1})	HA CQDs (cm^{-1})	Assignment
1	3670, 3545, 3279, and 3075	3950, 3441, 3194 and 3039	Hydroxy (OH) and amino (NH) stretching
2	2981, 2900 and 2861	2968, 2925 and 2886	Asymmetric and symmetric (C-H) stretching
3	-	2754 and 2624	Aldehyde (C-H) stretching
4	1626	1701	Carboxylic (C=O) stretching
5	1566	1587	Amide band
6	1444 and 1406	1465 and 1427	N-acetyl ($-COCH_3$) stretching
7	1374	1356	C-H bending
8	1231	1249	C-N stretching
9	1147	1176 and 1116	Glycosidic (ether) (C-O-C) group
10	1071 and 1031	1091 and 1050	Alcoholic (C-OH) group
11	995, 950 and 929	982	Proteoglycan sugar ring (C-O)
12	868, 792 ,742 685 and 626	799 and 761	C-H bending
